# Comparative Evaluation of Biomarkers of Inflammation Among Indian Women With Polycystic Ovary Syndrome (PCOS) Consuming Vegetarian vs. Non-vegetarian Diet

**DOI:** 10.3389/fendo.2019.00699

**Published:** 2019-11-08

**Authors:** Mohd Ashraf Ganie, Tajali Sahar, Aafia Rashid, Ishfaq A. Wani, Sobia Nisar, Thozhukat Sathyapalan, Sreenivas Vishnubhatla, Lakshmy Ramakrishnan, Tabasum Parvez, Ishaq Geer

**Affiliations:** ^1^Department of Endocrinology, Sheri-Kashmir Institute of Medical Sciences, Srinagar, India; ^2^Department of Medicine, Government Medical College, Srinagar, India; ^3^Department of Academic Endocrinology Diabetes and Metabolism, Hull York Medical School, University of Hull, Kingston upon Hull, United Kingdom; ^4^Departments of Biostatistics, All India Institute of Medical Sciences, New Delhi, India; ^5^Departments of Cardiac Biochemistry, All India Institute of Medical Sciences, New Delhi, India; ^6^Department of Obstetrics and Gynaecology, Sheri Kashmir Institute of Medical Sciences, Srinagar, India; ^7^Department of Pharmaceutical Sciences, University of Kashmir, Srinagar, India

**Keywords:** PCOS, inflammation, insulin resistance, testosterone, hs-CRP, adiponectin

## Abstract

**Background:** Sub-inflammation and insulin resistance characterize women with PCOS. Data on dietary modulation of inflammation among PCOS women is scant, particularly from Indian subcontinent. The present study aimed to assess the effect of plant based vs. animal origin diets on serum markers of inflammation (primary outcome measure).

**Methods:** This observational case-control study compared age and BMI matched PCOS and apparently healthy women from two populations following different dietary practices. The vegetarian women from New-Delhi (*n* = 82 PCOS and *n* = 179 healthy) and non-vegetarian women from Srinagar (*n* = 62 PCOS and *n* = 141 healthy) formed the groups. Using a uniform methodology, detailed clinical, biochemical, hormonal, and inflammatory marker assessment was undertaken.

**Results:** The mean age of the overall cohort was 26.23 ± 4.59 years with a mean BMI of 24.39 ± 3.72 kg/m^2^. Overall pro-inflammatory markers (TNF-α, IL-6, IL-1β, hs-CRP and serum resistin) were significantly higher (*p* ≤ 0.05) and anti-inflammatory markers (IL-10 and adiponectin) were lower among women with PCOS than healthy subjects. On comparing vegetarian women with non-vegetarians, higher daily calorie intake (1895.46 ± 258.19 vs. 1860.13 ± 323.96 Kcal) with a higher protein and fat and lower carbohydrate intake was recorded in the latter, although the percent energy derived from carbohydrates was higher among vegetarians. Clinical and biochemical parameters were comparable among the groups except mFG score, total serum testosterone and serum lipid levels which were higher among non-vegetarian women as compared to their vegetarian counterparts from both categories (PCOS and healthy). Interestingly, vegetarian women with PCOS and healthy women had higher serum pro-inflammatory and lower anti-inflammatory markers compared to their non-vegetarian counterparts.

**Conclusion:** Women with PCOS consuming Indian vegetarian diet have higher pro-inflammatory and lower anti-inflammatory marker levels than their age and BMI matched healthy non-vegetarian counterparts. This interesting observation can be attributed to the dietary composition, among other factors and needs confirmation from well-designed randomized studies on a larger cohort.

**Clinical Trial Registration:** The study was registered with CTRI database under registration number CTRI/2013/09/003996.

## Introduction

Polycystic ovary syndrome (PCOS), is a multifaceted disorder associated with a host of co-morbidities, including obesity, metabolic syndrome (MS), insulin resistance (IR), abnormal glucose tolerance (AGT), non-alcoholic fatty liver disease (NAFLD), psychiatric disturbances, elevated cardiovascular disease (CVD), and cancer risk etc. (1–6), in addition to several reproductive and cosmetic dysfunctions. The condition is known to affect 5–10% women of reproductive age in the West but is commoner in India with preliminary reports suggesting a prevalence as high as 22.5% (7, 8). Exact etiology being uncertain, the condition is characterized by two dominant pathogenic mechanisms namely hyperandrogenism and IR, both of which may lead to distinct clinical phenotypes and ovarian morphological patterns on ultrasound in women with PCOS (9). Chronic inflammation, a common accompaniment of these metabolic conditions, is characterized by elevation in pro-inflammatory cytokines, chemokines and markers of oxidative stress which in turn is linked to IR (10–13). Published data suggests higher levels of inflammatory markers or their gene polymorphisms among women with PCOS (14–16). Boulman et al. demonstrated elevated hs-CRP levels among insulin resistant women with PCOS similar to that observed by Mazibrada et al. (14, 17) while a few reports documented TNF-α and IL-6 gene polymorphisms among PCOS women in relation to hyperandrogenic phenotypic traits (15, 16). Although the reason of this sub-inflammation is unclear, higher BMI particularly visceral adiposity has been implicated (10, 18).

Dietary patterns have been reported to independently influence inflammatory and endothelial markers among healthy individuals (19, 20). Mediterranean diet, primarily based on sufficient intake of green vegetables, fruits, whole grains, sea food, and low red meat consumption (21) has been reported to have a beneficial effect upon development of type 2 diabetes mellitus (T2DM), inflammatory markers (such as IL-6, hs-CRP, and adiponectin), endothelial function and coagulation (20, 22–25). Similarly, vegetarian diets have been shown to lower lipid parameters (26) and hs-CRP (27). On the contrary, higher carbohydrate intake rather than high fat intake was associated with high total mortality in a recent large multinational epidemiological cohort (28). South Asians, particularly Indians have been demonstrated to have a higher prevalence of T2DM (29), CVD (30), PCOS (8) and sub-inflammation. These higher risks are partly attributed to dietary patterns, typically consisting of high percentage of carbohydrates and saturated fats from vegetables, rice, chapatis, or breads etc. (31). Whether the composition of Indian diet actually has a link to the epidemic of these disorders remains to be seen. In view of the paucity of data evaluating impact of Indian diet patterns on inflammatory markers among women with PCOS, we undertook this study assessing effect of plant based vs. animal origin diets on serum markers of inflammation as a primary outcome measure and clinical and metabolic parameters as secondary outcome measures.

## Materials and Methods

This cross sectional study recruited subjects from December 2014 to January 2018, from two cities located in North India- New Delhi and Srinagar. The women consuming plant based diets (vegetarian) form New Delhi and those consuming animal derived diets (non-vegetarian) from Srinagar, Jammu and Kashmir were eligible for the study. The study was conducted in accordance with the guidelines enshrined under the Helsinki 1975 declaration and was approved by the Ethics Committees of the respective study institutions. An informed written consent was taken from all the subjects before their enrolment.

### Subjects

All consecutive women (18–40 years) attending outpatient clinics of endocrinology and gynecology of All India Institute of Medical Sciences (AIIMS), New Delhi and Sheri-Kashmir Institute of Medical Sciences (SKIMS), Srinagar, Jammu and Kashmir for complaints of unwanted hair growth, irregular menstrual cycles and other symptoms of PCOS were informed about the study. Women who fulfilled Rotterdam 2003 criteria for the diagnosis of PCOS and volunteered to be part of the study were required to sign an informed consent. In order to remove the confounding of age and body weight on inflammatory markers and other metabolic parameters, the women were recruited in blocks of age (18–20, 21–25, 26–30, 31–35, 36–40 years) and BMI categories(<20, 20–25, >25 kg/m^2^). Study tools, methods of data capture, SOPs, investigator training, lab evaluation etc. followed a uniform protocol at both the centers.

### Clinical Assessment

All women were interviewed for details of their menstrual cyclicity (age of menarche, duration and number of cycles per year), features of hyperandrogenism (duration and extent of unwanted hair growth, acne vulgaris, and androgenic alopecia), weight gain, infertility, history of drug intake etc. as per the the pre-designed uniform questionnaire (Supplementary Table 2) at both the participating centers. Oligomenorrhea was defined as a cycle interval of >35 days or <8 cycles per year and amenorrhea as cessation of cycles for more than 6 months. A detailed diet review using a food frequency questionnaire (FFQ) and 72 h dietary recall undertaken by qualified and trained dieticians to quantify various dietary components using specially designed diet software (Diet Cal, Profound Tech solutions, New Delhi) at both centers. For purposes of the study, women who consumed meat/chicken/fish/egg at least 5 days a week at least for last 1 year were considered as non-vegetarian and those who strictly adhered to plant based diets were taken as vegetarians. Women refusing consent, consuming medications such as glucocorticoids, insulin sensitizers, anti-epileptics, NSAIDs etc. known to affect glucose tolerance, insulin sensitivity, inflammatory markers, pregnant women, or women with history suggestive of controlled or uncontrolled hyperprolactinemia or androgen-secreting tumors, Cushing's syndrome, thyroid dysfunction, non-classical congenital hyperplasia, diabetes or AGT, at the time of enrolment were excluded from the study. Other exclusions included any prior history (at least 2 weeks) of infection, trauma, surgery or significant stress such as exams, bereavement, psychiatric comorbidity etc. known to generate an inflammatory response.

Body weight, height, waist circumference were measured using standard calibrated instruments (SECA 213, Hamburg Germany) followed by a detailed systemic examination including measurement of blood pressure (Omron HEM7120). A mean of three readings was taken as the final value for these parameters. Quantitation of hirsutism using modified Ferriman-Gallwey (mFG) score (8 or above out of a total of 36 from nine body areas taken as significant), grading of acanthosis nigricans, acne vulgaris, and androgenic alopecia was done by a single observer at each center. The inter-observer variation between the FG scores among the trained observers at two centers was <4%.

### Laboratory Evaluation

After an overnight (10–12 h) fast all the participants were subjected to blood sampling arranged in the follicular phase (2nd to 7th day) of a spontaneous or medroxyprogesterone induced menstrual cycle. The samples were immediately separated in a cold centrifuge and aliquoted for biochemistry, hormones, and inflammatory markers. Biochemical parameters were assayed on the spot while the aliquots for hormones and inflammatory markers were stored at −80°C until the assay. The PCO morphology was assessed with trans-abdominal ultrasonography performed in the follicular phase by a single sonologist at each center using 7.5 mHz probe (AlokaSSD-500, Tokyo, Japan) to quantitate ovarian volume, count ovarian follicle number, and assess thecal hyper echogenicity with a common SOP.

### Controls

Apparently healthy, age, and BMI matched women were recruited from community clusters as part of health awareness-cum-screening outreach programmes conducted by the respective institutes. These women underwent similar clinical and laboratory evaluation as in cases.

### Assays

Biochemical parameters (plasma glucose, lipids, uric acid, calcium, phosphorus, liver and kidney function) were estimated using standard commercially available kits as per manufacturer's instructions on fully automated biochemical analysers (Hitachi 920, Japan). Samples for hormonal parameters (serum total T4, TSH, LH, FSH, PRL, cortisol,17OHP, total testosterone and insulin) from both the centers, were assayed using Electrochemiluminescence immunoassay (ECLIA) using Cobas e411(Roche Diagnostics Limited, USA). The inter- and intra-assay coefficients of variation were <7%. Serum inflammatory marker (TNF-α, IL-1β, IL-6, IL-10, hs-CRP, resistin, and adiponectin) levels were assayed by ELISA, using commercially available kits and according to supplier's protocol (Diaclone, France and Calbiotech, CA, USA). The inter- and intra-assay coefficients of variation were as per the manufacture prescriptions. Both hormonal and inflammatory markers were assayed at departmental laboratory of AIIMS New Delhi.

### Sample Size Calculation

Sample size was calculated using G^*^Power software (version 3.1.9.2). Considering type one error (α) as 0.05, power of study as 90% and effect size 0.3 with reference from a recent study comparing the inflammatory profile of vegetarians and omnivores (32), a minimum of 50 subjects were required per group. Therefore, to account for non-response and incompleteness of data, we planned to recruit a minimum number of 62 cases in each group and 124 controls in each group with case to control ratio of 2:1.

### Statistical Analysis

The Statistical Package for Social Sciences-22 software was used for statistical analysis (SPSS Inc., Chicago, IL, USA). Data has been depicted as mean ± standard deviation and was log transformed, wherever necessary. For assessment of normality, Kolmogorov Smirnov test was employed. One way ANOVA was used for comparing more than two groups and Mann-Witney *U*-test were used for comparing two groups. Parameters with *p* ≤ 0.05 were considered as statistically significant.

## Results

**Figure 1:**
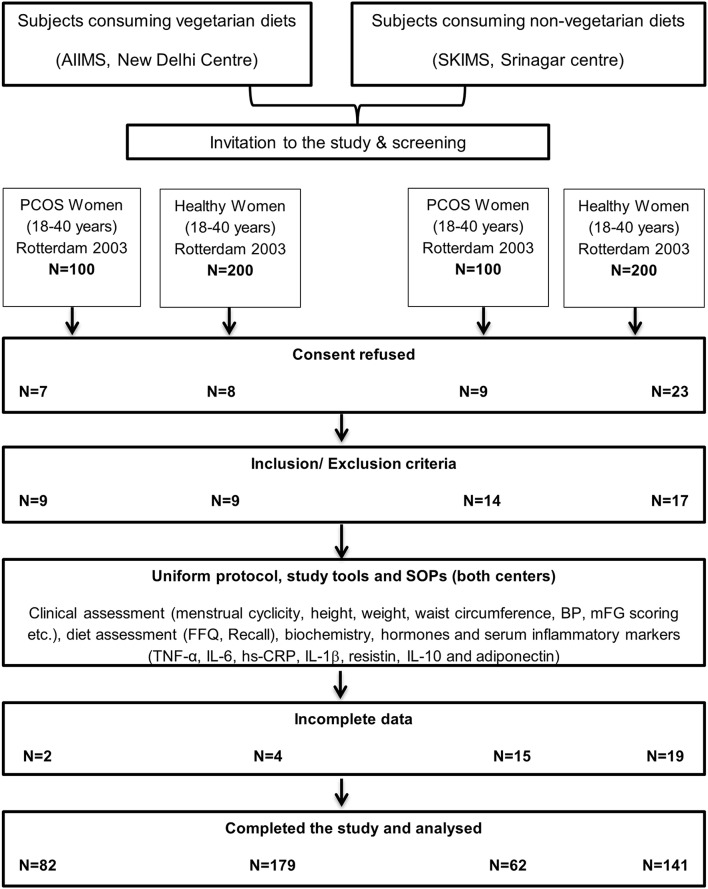
Showing PERT chart describing the flow of subjects throughout this study.

A total of 200 women, qualifying Rotterdam 2003 for the diagnosis of PCOS, were screened (*n* = 100 at each center) out of which 62 non-vegetarian (from Srinagar) and 82 vegetarian (from Delhi) consented and had complete data for analysis. Another 400 apparently healthy age and BMI matched women (*n* = 200 at each center) were screened and invited to participate as controls out of which a total of 320 women (179 vegetarian women from Delhi and 141 non-vegetarian women from Srinagar) consented and had complete data available for analysis (Figure 1). Group-wise comparisons of their clinical, biochemical, hormonal and inflammatory marker profiles are shown in Tables 1–3.

**Table 1:** Showing comparison of clinical and hormonal parameters among PCOS vs. healthy women from Delhi and Srinagar.

**Parameters**	**Vegetarian group**		**Non-vegetarian group**					
	**Women with PCOS** **(*n* = 82)** **Mean ± SD**	**Healthy women** **(*n* = 179)** **Mean ± SD**	**Women with PCOS** **(*n* = 62)** **Mean ± SD**	**Healthy women** **(*n* = 141)** **Mean ± SD**	***p*-value** **(a =)**	***p*-value** **(b =)**	***p*-value** **(c =)**	***p*-value** **(d =)**
Age (years)	25.68 ± 3.81	26.53 ± 5.99	26.13 ± 4.43	26.57 ± 4.11	0.06	0.59	0.53	0.55
No. of menstrual cycles/year	8.02 ± 2.38	11.72 ± 0.65^a^	8.13 ± 3.40	11.99 ± 0.15^d^	< 0.01	0.705	0.14	< 0.01
Ferriman–Gallwey score (mFG)	11.32 ± 4.52	5.51 ± 2.77^a^	11.82 ± 4.23	6.25 ± 0.76^c^^,^ ^d^	< 0.01	0.43	0.03	< 0.01
BMI (Kg/m^2^)	24.94 ± 3.61	23.96 ± 4.17	24.68 ± 3.45	23.97 ± 3.63	0.12	0.467	0.10	0.07
Waist circumference (cm)	83.39 ± 11.49	82.29 ± 12.53	83.42 ± 8.81	83.62 ± 10.94	0.20	0.98	0.09	0.91
Systolic blood pressure (mmHg)	115.49 ± 13.19	115.58 ± 14.51	116.77 ± 8.64	113.43 ± 10.01	0.96	0.53	0.14	0.08
Diastolic blood pressure (mmHg)	77.38 ± 10.08	77.38 ± 10.48	78.15 ± 6.79	76.33 ± 8.78	0.99	0.63	0.34	0.21
Serum total T4 (μg/dl)	8.78 ± 2.23	8.41 ± 1.98	8.27 ± 1.59	8.12 ± 2.12	0.18	0.15	0.22	0.63
Serum TSH (μIU/ml)	3.25 ± 1.81	3.22 ± 1.69	3.45 ± 1.68	3.35 ± 1.39	0.88	0.46	0.48	0.70
Serum prolactin (ng/ml)	16.47 ± 5.38	16.06 ± 4.97	16.17 ± 5.92	15.15 ± 4.25	0.54	0.72	0.11	0.18
Serum LH (IU/ml)	7.75 ± 3.58	6.26 ± 2.36^a^	7.42 ± 3.56	6.59 ± 2.39^d^	< 0.01	0.48	0.31	0.06
Serum FSH (IU/ml)	6.08 ± 2.02	6.99 ± 2.43^a^	6.30 ± 2.13	7.16 ± 1.94^d^	< 0.01	0.51	0.48	< 0.01
Serum total testosterone (ng/ml)	0.48 ± 0.29	0.22 ± 0.18^a^	0.56 ± 0.25^b^	0.31 ± 0.08^c^^,^^d^	< 0.01	< 0.01	0.01	< 0.01
Serum 25OHD (ng/ml)	11.66 ± 9.81	15.83 ± 8.80^a^	11.24 ± 6.58	15.74 ± 7.23^d^	0.03	0.72	0.63	< 0.01
Protein (g/day)	50.63 ± 11.48	52.02 ± 16.62	55.16 ± 12.34^b^	54.19 ± 11.03^c^	0.87	0.05	0.05	0.23
%Energy from proteins	10.97 ± 2.04	11.06 ± 2.91	11.47 ± 2.41	11.49 ± 2.26				
Fat (g/day)	43.11 ± 14.72	42.05 ± 16.93	49.07 ± 12.35^b^	46.10 ± 9.46^c^	0.09	< 0.01	< 0.01	0.15
%Energy from fat	20.81 ± 6.03	19.91 ± 6.65	23.11 ± 5.66^b^	21.97 ± 4.37^c^				
Carbohydrate (g/day)	318.24 ± 53.94	313.83 ± 72.87	301.86 ± 58.45^b^	304.93 ± 44.16^c^	0.07	< 0.01	< 0.01	0.54
%Energy from carbohydrates	65.13 ± 7.78	66.94 ± 7.84	62.02 ± 9.12^b^	64.24 ± 5.54^c^				
Energy (Kcal/day)	1862.78 ± 262.33	1857.47 ± 385.59^a^	1895.51 ± 308.28^b^	1895.40 ± 208.11^c^	0.04	< 0.01	< 0.01	0.68

a *p < 0.05 vegetarian PCOS vs. healthy vegetarian women*,

b *p < 0.05 vegetarian PCOS vs. non-vegetarian PCOS*,

c *p < 0.05 healthy vegetarian women vs. healthy non-vegetarian*,

d *p < 0.05 non-vegetarian PCOS vs. non-vegetarian healthy women. For assessment of normality, Kolmogorov Smirnov test was employed*.

*Values are presented as mean ± standard deviation. P-values were calculated using one-way ANOVA followed by multiple comparisons [post hoc test (Least Significance Difference (LSD))]*.

**Table 2:** Showing comparison of biochemical parameters among healthy women and women with PCOS from Delhi and Srinagar.

**Parameters**	**Vegetarian group**		**Non-vegetarian group**					
	**Women with PCOS** **(*n* = 82)** **Mean ± SD**	**Healthy women** **(*n* = 179)** **Mean ± SD**	**Women with PCOS** **(*n* = 62)** **Mean ± SD**	**Healthy women** **(*n* = 141)** **Mean ± SD**	***p*-value** **(a =)**	***p*-value** **(b =)**	***p*-value** **(c =)**	***p*-value** **(d =)**
Blood glucose- fasting (mg/dl)	87.71 ± 10.53	86.55 ± 10.34	86.57 ± 10.70	83.37 ± 9.13^c^^,^ ^d^	0.91	0.51	< 0.01	0.03
Fasting plasma insulin-(mIU/ml)	13.07 ± 8.62	9.45 ± 8.99^a^	12.06 ± 5.91	7.73 ± 3.52^c^^,^^d^	< 0.01	0.41	0.03	< 0.01
HOMA-IR	2.77 ± 1.93	1.72 ± 1.23^a^	2.57 ± 1.24	1.74 ± 1.27^d^	< 0.01	0.41	0.93	< 0.01
QUICKI	0.36 ± 0.09	0.37 ± 0.03^a^	0.34 ± 0.03^b^	0.37 ± 0.04^d^	0.03	0.01	0.52	< 0.01
FGIR	9.12 ± 4.71	14.56 ± 9.21^a^	8.98 ± 4.82	15.87 ± 11.93^d^	0.02	0.94	0.22	< 0.01
Serum urea (mg/dl)	22.12 ± 5.62	23.55 ± 6.84	23.72 ± 4.57	23.77 ± 5.12	0.16	0.14	0.51	0.78
Serum creatinine (mg/dl)	0.65 ± 0.12	0.65 ± 0.17	0.72 ± 0.17^b^	0.78 ± 0.21^c^	0.85	0.03	< 0.01	0.23
Serum total calcium (mg/dl)	9.02 ± 0.73	8.43 ± 1.16	9.24 ± 0.63^b^	8.93 ± 0.75^d^	0.41	0.13	0.26	< 0.01
Serum phosphate (mg/dl)	3.59 ± 0.92	3.84 ± 1.27	4.02 ± 0.89	4.05 ± 0.95	0.26	0.41	0.16	0.58
Serum uric acid (mg/dl)	4.3 ± 1.03	4.13 ± 0.96	4.53 ± 0.82	4.18 ± 0.98	0.07	0.19	0.18	0.06
Serum bilirubin (mg/dl)	0.73 ± 0.23	0.72 ± 0.44	0.79 ± 0.34	0.76 ± 0.34	0.31	0.13	0.41	0.53
Serum SGOT (IU/L)	24.78 ± 8.61	23.72 ± 11.33^a^	23.72 ± 7.21	22.9 ± 8.59^d^	0.02	0.07	0.11	0.05
Serum alkaline phosphatase (IU/L)	107.04 ± 32.16	101.31 ± 33.72^a^	105.71 ± 28.52	94.84 ± 24.3^c^^,^^d^	0.03	0.11	< 0.01	< 0.01
Serum total protein (g/dl)	7.34 ± 0.39	7.62 ± 0.59^a^	8.18 ± 0.54b	7.98 ± 1.04^c^^,^^d^	< 0.01	0.01	0.05	< 0.01
Serum albumin (g/dl)	4.45 ± 0.43	4.39 ± 0.60	4.55 ± 0.27	4.66 ± 0.51	0.08	0.20	0.12	0.19
Serum total cholesterol (mg/dl)	163.95 ± 32.69	156.08 ± 33.43	168.84 ± 36.62	165.56 ± 34.39^d^	0.08	0.31	0.14	0.02
Serum HDL cholesterol (mg/dl)	46.90 ± 15.59	46.65 ± 12.22	46.62 ± 16.28	48.89 ± 17.75	0.90	0.91	0.18	0.33
Serum LDL cholesterol (mg/dl)	94.4 ± 31.08	85.27 ± 25.48^a^	104.32 ± 24.14^b^	92.48 ± 25.27^c^^,^^d^	0.04	0.03	0.02	< 0.01
Serum Triglyceride (mg/dl)	105.18 ± 43.81	101.95 ± 37.15^a^	126.80 ± 39.04^b^	121.63 ± 49.67^c^	0.05	< 0.01	< 0.01	0.07

*Values are presented as mean ± standard deviation. p-values were calculated using one-way ANOVA followed by multiple comparisons [post hoc test (Least Significance Difference (LSD))]*.

a *p < 0.05 vegetarian PCOS vs. healthy vegetarian women*,

b *p < 0.05 vegetarian PCOS vs. non-vegetarian PCOS*,

c *p < 0.05 healthy vegetarian women vs. healthy non-vegetarian*,

d *p < 0.05 non-vegetarian PCOS vs. non-vegetarian healthy women*.

**Table 3:** Showing comparison of serum biomarkers of inflammation among women with PCOS and healthy controls from Delhi and Srinagar.

**Parameter**	**Vegetarian group**		**Non-vegetarian group**					
	**Women with PCOS (*n* = 82)** **Mean ± SD**	**Healthy women (*n* = 179)** **Mean ± SD**	**Women with PCOS (*n* = 62)** **Mean ± SD**	**Healthy women (*n* = 141)** **Mean ± SD**	***p*-value (a =)**	***p*-value (b =)**	***p*-value (c =)**	***p*-value (d =)**
Serum TNF-α (pg/ml)	45.13 ± 35.74	24.18 ± 20.18^a^	35.47 ± 23.68	22.98 ± 16.42^d^	0.03	0.91	0.21	< 0.01
Serum IL-6 (pg/ml)	22.95 ± 14.40	8.44 ± 5.64^a^	19.86 ± 7.85	4.39 ± 3.94^c^^,^ ^d^	< 0.01	0.91	< 0.01	< 0.01
Serum IL-1β (pg/ml)	11.85 ± 7.43	8.55 ± 5.9^a^	9.79 ± 5.12	7.52 ± 4.35^d^	< 0.01	0.43	0.37	0.02
Serum hs-CRP (ng/ml)	3.83 ± 1.68	2.19 ± 1.48^a^	2.38 ± 0.88^b^	1.68 ± 1.52^c^^,^^d^	< 0.01	0.01	< 0.01	< 0.01
Serum resistin (ng/ml)	10.84 ± 4.54	6.18 ± 3.69^a^	6.27 ± 2.28^b^	5.48 ± 3.10	< 0.01	< 0.01	0.16	0.03
Serum adiponectin (ng/ml)	3.15 ± 2.01	6.75 ± 4.45^a^	6.33 ± 2.81^b^	7.20 ± 5.38^c^^,^^d^	< 0.01	< 0.01	0.05	0.02
Serum IL-10(pg/ml)	6.47 ± 1.93	9.46 ± 3.60^a^	6.71 ± 3.16	10.53 ± 7.47^d^	< 0.01	0.68	0.24	< 0.01

*Values are presented as mean ± standard deviation. P-values were calculated using Mann-Whitney U test*.

a *p < 0.05 vegetarian PCOS vs. healthy vegetarian women*,

b *p < 0.05 vegetarian PCOS vs. non-vegetarian PCOS*,

c *p < 0.05 healthy vegetarian women vs. healthy non-vegetarian*,

d *p < 0.05 non-vegetarian PCOS vs. non-vegetarian healthy women. For assessment of normality, Kolmogorov Smirnov test was employed*.

### Comparison Between Women With PCOS and Healthy Controls

The overall respective mean age of PCOS subjects (*n* = 144) and controls (*n* = 320) was 26.06 ± 4.12 vs. 26.55 ± 5.05 years while as their mean BMI was 24.81 ± 3.53 vs. 23.97 ± 3.90 kg/m^2^. The mean number of menstrual cycles per year (8.10 ± 2.83 vs. 11.86 ± 2.88) was significantly lower while as mFG scores (11.57 ± 4.36 vs. 5.88 ± 1.77), serum LH (7.58 ± 3.57 vs. 6.42 ± 2.37 IU/ml) and serum total testosterone (0.52 ± 0.27 vs. 0.27 ± 0.13 ng/ml) levels were significantly higher among women with PCOS as compared to healthy women from both the centers. Plasma 25OHD levels were marginally lower (11.45 ± 8.19 vs. 15.78 ± 8.02 ng/ml) among women with PCOS (Supplementary Table 1). Fasting plasma insulin (12.57 ± 7.27 vs. 8.59 ± 6.26 mIU/ml), HOMA-IR (2.67 ± 1.58 vs. 1.73 ± 1.15) were higher while as FGIR (9.05 ± 4.76 vs. 15.21 ± 10.57) and QUICKI (0.35 ± 0.06 vs. 0.37 ± 0.04) were significantly lower among PCOS women than healthy controls. Pro-inflammatory markers (TNF-α, IL-6, IL-1β, hs-CRP, and serum resistin) were significantly higher (*p* ≤ 0.05) and anti-inflammatory markers (IL-10 and adiponectin) significantly lower among women with PCOS than their healthy counterparts (Supplementary Table 1). Other parameters like waist circumference, blood pressure, uric acid and serum phosphorous did not differ significantly among the groups.

### Comparison Between Vegetarian and Non-vegetarian Women With PCOS

Age (25.68 ± 3.81 vs. 26.13 ± 4.43 years) and BMI (24.94 ± 3.61 vs. 24.68 ± 3.45 kg/m^2^) matched vegetarian and non-vegetarian PCOS women were comparable with regard to most of the clinical (mean number of menstrual cycles per year, mFG score, BP), biochemical (mean plasma glucose, HOMA-IR, urea, uric acid, SGOT) and hormonal (serum LH, FSH, PRL) parameters. Biochemical parameters such as serum creatinine, serum triglycerides and LDL cholesterol levels were higher (*p* ≤ 0.05) among the non-vegetarian PCOS women as compared to their vegetarian counterparts. Interestingly, hirsutism scores and serum total testosterone levels were higher among PCOS women from Srinagar (Tables 1–3). Another interesting observation was that the pro-inflammatory markers (serum hs-CRP, TNF-α, IL-6, and IL-1β) were elevated and anti-inflammatory (serum adiponectin and IL-10) were lower among vegetarian women with PCOS as compared to non-vegetarian women with PCOS, although statisticalyl significant difference was found only in cases of serum hs-CRP, resistin and adiponectin levels (Table 3). A comparison of macronutrient intake between two PCOS groups showed a higher self-reported daily calorie intake among non-vegetarian women (1895.51 ± 308.28 vs. 1862.78 ± 262.33 Kcal, *p* ≤ 0.05) with higher daily fat (49.07 ± 12.35 g vs. 43.11 ± 14.72 g) and protein intake (55.16 ± 12.34 g vs. 50.63 ± 11.48 g) and a lower carbohydrate intake (301.86 ± 58.45 vs. 318.24 ± 53.94 g) than their vegetarian counterparts (*p* ≤ 0.05).

### Comparison Between Healthy Vegetarian and Non-vegetarian Women

Most of the clinical, biochemical, and hormonal parameters were comparable among the subgroups (vegetarian vs. non-vegetarian) of healthy women. Few exceptions were mFG score, serum creatinine, TG, LDL cholesterol levels and total testosterone levels, which were higher among healthy non-vegetarian women from Srinagar, similar to the trend found in women with PCOS subgroups. Healthy vegetarian women from Delhi had higher mean fasting plasma glucose and insulin levels. Similar to the observations in PCOS subgroups, pro-inflammatory markers were higher and anti-inflammatory markers were lower among the healthy vegetarian women from Delhi. However, again this attained statistical significance only in cases of serum hs-CRP, IL-6, and adiponectin. A comparison of macronutrient intake between two healthy groups showed a higher self-reported daily calorie intake among non-vegetarian women (1895.40 ± 208.11 vs. 1857.47 ± 385.59 Kcal, *p* ≤ 0.05) with higher daily fat (46.10 ± 9.46 vs. 42.05 ± 16.93 g) and protein intake (54.19 ± 11.03 vs. 52.02 ± 16.62 g) and a lower carbohydrate intake (304.93 ± 44.16 vs. 313.83 ± 72.87 g; *p* ≤ 0.05) than their vegetarian counterparts.

On comparing the per cent energy consumption from macronutrients, vegetarian women consumed lower energy from proteins (11.01 ± 2.22% vs. 11.48 ± 2.34%) and fats (20.36 ± 6.34 vs. 22.54 ± 5.05%) (*p* ≤ 0.05) and higher energy from carbohydrates (66.04 ± 7.82% vs. 63.13 ± 7.32%) (*p* ≤ 0.05) as compared to non-vegetarian women in our cohort.

**Figure 2:**
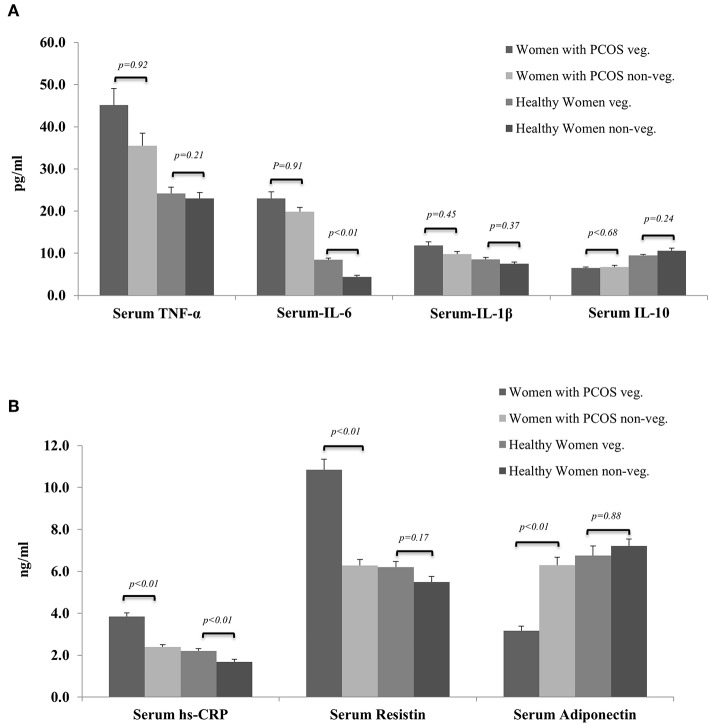
Showing comparison of serum levels of biomarkers of inflammation among women with PCOS and healthy controls from vegetarian and non-vegetarian backgrounds. **(A)** Serum TNF-α, IL-6, IL-1b, and IL-10. **(B)** Serum hs-CRP, resistin, and adiponectin.

Figure 2 shows overall trends of serum inflammatory marker profiles among healthy and PCOS women from both vegetarian and non-vegetarian subgroups. Overall the pro-inflammatory markers (TNF-α, IL-6, IL-1β, hs-CRP, and serum resistin) were highest among vegetarian women with PCOS followed by age and BMI matched non-vegetarian women with PCOS which was in turn were higher than healthy vegetarian women. The lowest levels of pro-inflammatory markers were observed in the healthy non-vegetarian women. A similar but reverse trend was observed in anti-inflammatory markers (IL-10 and adiponectin) among these subgroups.

## Discussion

In the present study, we aimed to compare the inflammatory biomarker profiles of Indian women with PCOS following vegetarian and non-vegetarian dietary patterns from two different centers in North India. The key results revealed higher pro-inflammatory markers (TNF-α, IL-6, IL-1β, resistin and hs-CRP) among women with PCOS from both the centers as compared to age and BMI matched healthy controls from the respective populations. On comparing inflammatory markers among the PCOS subgroups pursuing different dietary patterns, vegetarian women had higher levels of serum pro-inflammatory (TNF-α, IL-6, hs-CRP, resistin and IL-1β) and lower levels of anti-inflammatory markers (serum IL-10 and adiponectin) reaching statistical significance in case of serum hs-CRP, resistin and adiponectin. A similar trend was observed when healthy control groups from two populations were compared.

However, the study results can furnish limited inferences owing to some limitations such as lack of data on micronutrients including vitamin B12, omega-3 fatty acids, quantitation of visceral fat mass and weighing of lifestyle differences such as pollution (pollution index of Delhi vs. Srinagar: 91.74 vs. 35.01), stress levels etc. among the two populations. Although, it would have been advantageous to enroll both vegetarian and non-vegetarian women from each center, it was not feasible to enroll pure vegetarian women from the Srinagar centre, since the population is habitually non-vegetarian. Nevertheless, this is the first study reporting impact of diet on inflammatory markers among women with PCOS with a reasonable sample size and age and BMI matched control groups.

Low grade chronic inflammation also referred to as inflammaging is incriminated in the aetiopathogenesis of many chronic illnesses, notably metabolic syndrome (10, 13), obesity (10), T2DM (33), CAD (34), neurodegenerative diseases (35), reproductive dysfunctions including PCOS (13, 14, 17, 36). There is paucity of data on diet-induced inflammation among Asians in general and Indian women with PCOS in particular. Therefore, we undertook this study to evaluate the impact of vegetarian vs. non-vegetarian diet on cytokines among North Indian women with PCOS.

As expected PCOS women (from both centers) had less number of menstrual cycles per year, had more severe hirsutism, elevated serum total testosterone, fasting plasma glucose, and insulin levels as compared to healthy controls, which is in accordance with previously published literature (3, 5, 18). Serum 25OHD levels were lower, while serum alkaline phosphatase was higher among women with PCOS. This could be attributed to their increased body fat content especially the visceral adiposity as reported earlier (37, 38).

We observed that most of the clinical, biochemical, and hormonal parameters were comparable between PCOS women from both centers (vegetarian vs. non-vegetarian) except serum LDL cholesterol, triglycerides and creatinine which were higher among non-vegetarians. This can be attributed to higher daily fat and protein intake among the non-vegetarian subgroups and the observation is supported by previous studies suggesting that plant based diets have beneficial effects on serum lipid levels (26, 39). The intriguing finding of higher mFG scores and serum total testosterone among healthy as well as women with PCOS from Srinagar as compared to their age and BMI-matched women from Delhi is not readily explainable. However, this finding is consistent with our previously published data (40) and can be attributed to ethnic differences among the two populations. This finding of higher androgen levels among women from Srinagar centre may be one of the reasons of better inflammatory marker profile although the earlier data analyzing the impact of androgens on inflammation is contradictory and needs further evaluation (41).

The observation of a higher pro-inflammatory state among women with PCOS compared to healthy controls is similar to earlier studies showing higher hs-CRP levels among women with PCOS than healthy controls (14, 17). Although, a systematic review and meta-analysis by Escobar-Morreale et al. (42) negated any difference in the levels of serum IL-6 and TNF-α between women with PCOS and controls, this meta-analysis had some limitations. They included 10 studies enrolling small number of PCOS subjects (*n* = 523) and controls (*n* = 330) with heterogeneous backgrounds from different ethnicities while as we had relatively homogenous population with fair number of subjects in the present study.

Interestingly, among the PCOS subgroups, vegetarian women had significantly higher levels of serum hs-CRP and serum resistin and lower levels of anti-inflammatory adiponectin. Although, there is no data evaluating the impact of different diets among women with PCOS globally, these findings seem to be in contradiction to most previous studies showing lower inflammatory marker levels among non-PCOS subjects following plant-based diets (19, 32, 42). Nettleton et al. in a multi-ethnic study showed that vegetarian diet comprising of whole grains, fruits and green leafy vegetables was inversely related to serum hs-CRP and IL-6 concentrations (19). Randomized studies have shown that a traditional Mediterranean diet is highly beneficial in lowering inflammation (43–45). In a recent meta-analysis of 136,846 participants, Koloverou et al. observed that strict adherence to Mediterranean diet reduces the risk of developing type 2 DM by 23% (25). Although, a minority of reports among non-PCOS subjects support our observations (46, 47), to the best of our knowledge this is the first study evaluating the impact of diet on inflammatory markers in PCOS and healthy women. There is also suggestion that strict adherence to vegetarian diets without necessary supplementation adversely affects female fertility outcomes in women (48, 49). This higher inflammaging among women (both healthy and PCOS) pursuing vegetarian diet in the present study is unlikely to be due to higher fat mass or higher age as our women were age and BMI matched. This could be attributed to differences in dietary composition (micronutrient or macronutrient) or the methods of preparation of Indian vegetarian diet as opposed to Mediterranean diet. Unlike Mediterranean diet, Indian vegetarian diet is generally rich in carbohydrates and low in omega-3 fatty acids (50) which could also be an explanation for a higher pro-inflammatory marker elevation. A recent cohort study conducted across eighteen countries with participants from different ethnicities by Dehghan et al. found that a higher total fat intake was associated with lower risk of total mortality, cardiovascular diseases and stroke (28). In line with this study, we observed that the vegetarian subjects, despite having lower overall daily calorie intake, recorded a higher per cent energy intake from carbohydrates and lower intake from fats as compared to non-vegetarian subjects and may partly explain the pro-inflammatory state in them. Our results are also supported by recent evidence correlating vegetarian diet with poor health status among Asian Indians in which vegetarianism was found to be associated with higher incidence of metabolic syndrome and obesity (51).

A surge in prevalence of metabolic syndrome, obesity, T2DM, CAD etc. among Asian Indians, despite having lower BMIs has been reported (8, 30, 52) in recent past. The exact etiology being unknown, diet has been incriminated as one of the prime factors in the rising prevalence of these conditions. There is paucity of data on diet-induced inflammation among Asians in general and Indian women with PCOS in particular. Our data is in contravention to earlier studies and traditional advice given to patients, although the implications of this study are not immediately utilizable in clinical practice and may be only generalizable after well-designed, larger long term studies are conducted to reproduce the findings.

In conclusion, this study reports higher inflammatory markers among women with PCOS as compared to healthy controls and the Indian vegetarian diet has adverse impact on this profile.

## Data Availability Statement

The datasets generated for this study are available on request to the corresponding author.

## Ethics Statement

The studies involving human participants were reviewed and approved by Institutional ethics committee-All India Institute of Medical Sciences, New Delhi and Institutional ethical committee- Sher-i-Kashmir Institute of Medical Sciences, Srinagar. The patients/participants provided their written informed consent to participate in this study. Written informed consent was obtained from the individual(s) for the publication of any potentially identifiable images or data included in this article.

## Author Contributions

MG, LR, SV, and TP designed the research. MG, TSah, and SN analyzed the data and wrote the manuscript. AR and IW conducted the research. TSat and IG also contributed in writing the paper. All authors approved the final content of the paper.

### Conflict of Interest

The authors declare that the research was conducted in the absence of any commercial or financial relationships that could be construed as a potential conflict of interest.
