# Supplementary material for: Comparative Evaluation of Biomarkers of Inflammation Among Indian Women With Polycystic Ovary Syndrome (PCOS) Consuming Vegetarian vs. Non-vegetarian Diet
Source: Front Endocrinol (Lausanne). 2019 Nov 8;10:699. doi: 10.3389/fendo.2019.00699 (PMC6857098; doi:10.3389/fendo.2019.00699)
Supplement: Supplementary file 1 [file Table_1.DOCX]

**Supplementary Table 1: Comparison between women with PCOS and healthy controls**

| **Parameters** | **Women with PCOS**  **(n=144)**  **Mean** ± **SD** | **Healthy women**  **(n=320)**  **Mean** ± **SD** | **p-value** |
| --- | --- | --- | --- |
| Age (years) | 26.06+4.12 | 26.55+5.05 | 0.10 |
| No. of menstrual cycles/year | 8.10±2.83 | 11.86±2.88 | <0.01 |
| Ferriman–Gallwey score (mFG) | 11.57±4.37 | 5.88±1.77 | <0.01 |
| BMI (Kg/m^2^) | 24.81±3.53 | 23.97±3.90 | 0.11 |
| Serum LH (IU/ml) | 7.58±3.57 | 6.42±2.37 | <0.01 |
| Serum FSH (IU/ml) | 6.19±2.07 | 7.07±2.19 | <0.01 |
| Serum total testosterone (ng/ml) | 0.52±0.27 | 0.27±0.13 | <0.01 |
| Serum 25OHD (ng/ml) | 11.45±8.19 | 15.78±8.02 | 0.05 |
| Blood glucose- fasting (mg/dl) | 87.14±10.61 | 84.96±9.36 | 0.12 |
| Fasting plasma insulin-(mIU/ml) | 12.57±7.27 | 8.59±6.26 | <0.01 |
| HOMA-IR | 2.67±1.58 | 1.73±1.25 | <0.01 |
| QUICKI | 0.35±0.06 | 0.37±0.04 | 0.02 |
| FGIR | 9.05±4.76 | 15.21±10.57 | <0.01 |
| Serum TNF-α (pg/ml) | 40.97±31.40 | 23.65±18.60 | <0.01 |
| Serum IL-6 (pg/ml) | 21.62±12.09 | 6.65±5.35 | <0.01 |
| Serum IL-1β (pg/ml) | 10.96±6.60 | 8.10±5.30 | <0.01 |
| Serum hs-CRP (ng/ml) | 3.21±1.56 | 1.96±1.52 | <0.01 |
| Serum resistin (ng/ml) | 8.88±4.36 | 5.88±3.46 | <0.01 |
| Serum adiponectin (ng/ml) | 4.52±2.85 | 6.95±4.88 | <0.01 |
| Serum IL-10(pg/ml) | 6.57±2.53 | 9.92±5.66 | <0.01 |

Values are presented as mean ± standard deviation. P values were calculated using independent sample t-test. A p value of ≤ 0.05 was considered as significant.
