# Supplementary material for: Comparative Evaluation of Biomarkers of Inflammation Among Indian Women With Polycystic Ovary Syndrome (PCOS) Consuming Vegetarian vs. Non-vegetarian Diet
Source: Front Endocrinol (Lausanne). 2019 Nov 8;10:699. doi: 10.3389/fendo.2019.00699 (PMC6857098; doi:10.3389/fendo.2019.00699)
Supplement: Supplementary file 2 [file Table_2.PDF]

DATE

SUBJECT ID:

INSTRUCTIONS: This questionnaire contains nine sections and will be administered to the female subject by the interviewer.

School/ College:	
Class:	
Section/ Specialization:	
Has consent been taken?	1 ☐ Yes 2 ☐ No
24 Hour Dietary Recall Status	Day 1 ☐ Day 2 ☐ Day 3 ☐
Semi Quantitative Food Frequency Questionnaire Status	1 ☐ Complete 2 ☐ Incomplete

Section 1: Background Information

Q.No.	Question	Response	Comments
1.	Name of the interviewee		
2.	Date of Birth		
3.	Age		
4.	Residential Address		
5.	Contact number	Mobile- Landline-	
6.	Email id		
7.	Type of Family	1 ☐ Nuclear 2 ☐ Joint 3 ☐ xtended<br>17 ☐ Any Other (Please Specify)_________________	
8.	Total family income (per month) (INR)	1 ☐ <25,000 2 ☐ 25,001 – 50,000 3 ☐ 50,001 – 75,000<br>4 ☐ 00-1,00,00 5 ☐ > 1, 00,000	
9.	Product of Consanguineous marriage	1. ☐ yes 2. ☐ No	
10	Family History of Type 2 diabetes Mellitus?	1. ☐ Yes 2. ☐ No	
11.	Last Menstrual Period (LMP)	DD _______ MM _______ YY _______________	

Section 1.1: IF YOU HAVE ANY OF THE FOLLOWING PROBLEMS*……………………… *PLEASE INFORM

- Did you have any prolonged illness or acute illness in last 2 weeks? Y/N
- Acute infection in last 6 weeks Y/N
- Examination or class assessment in the following or preceding week Y/N
- Known autoimmune disorders in self or family Y/N
- Chronic renal disease/Chronic liver disease/Known heart disease Y/N
- Diabetes mellitus /Hypertension/COPD/Chronic lung disease. Y/N
- Pregnancy and lactation in last 6 months Y/N
- Hypothyroidism /Goitre/thyroid surgery Y/N
- Drug intake (steroid, lithium, NSAIDs, ACE inhibitors, statins)-6 months Y/N
- Protein loss by kidney Y/N
- Neuromuscular land genetic illnesses Y/N
- Psychiatric disorder -6 months Y/N

Section 2: Diagnosis for Polycystic Ovary Syndrome

A.	Age of Menarche	________ Years
A.1	Menstrual cyclicity- Are your cycles regular?	1 ☐ Yes 2 ☐ No
A.2	If regular, what is the inter-menstrual interval? (Time from the first day of one period to the first day of the next period)	__________days
A.3	If irregular, how many cycles do you have in one year?	
A.4	What is the duration? AND<br><br>Amount of bleeding	__________days<br><br>___________number of pads/ day
B.	Do you have ANY unwanted (dark, coarse) hair anywhere on the body?	1 ☐ Yes 2 ☐ No
B.1.	If yes, kindly select the areas and the density they have?	(Tick on the figure overleaf)
B.2.	Duration Of Hirsutism	_____________ years
C.	Did you/ do you have acne (PIMPLES) anywhere on the body?	1 ☐ Yes 2 ☐ No
C.1.	If yes, how severe are/were they?	1 ☐ Mild 2 ☐ Moderate<br><br>3 ☐ Severe 4 ☐ Resistant to treatment
D.	Do you have any loss of scalp hair?	1 ☐ Yes 2 ☐ No
E.	Have you noticed that you have oily skin?	1. ☐ Yes 2. ☐ No

F.	Have you noticed dark patches on your skin, skin tags or tiny excess flaps of skin (neck, armpits, etc.)?	1. ☐ Yes 2. ☐ No			
G.	**Family History**				
.	Does anyone in your family (1st/ 2nd degree relative) have any of these?			Yes	No
		a.	Severe acne		
		b.	Menstrual disturbances		
		c.	Abnormal hair growth		
		d.	Diabetes mellitus (High blood sugar)		
		e.	Hypertension (High blood pressure)		
		f.	Infertility (female)		
		g.	Gout		
		h.	Early coronary artery disease/ heart attacks		
		i.	Breast cancer		
		j.	Uterine cancer		
		k.	Obesity		
		l.	Hypothryroidism		
F.	Do you take any medications?	1 ☐ Yes 2 ☐ No			
F.1.	If yes, what is the NAME of the medicine?				

Modified Ferriman-Gallwey Hirsutism Score

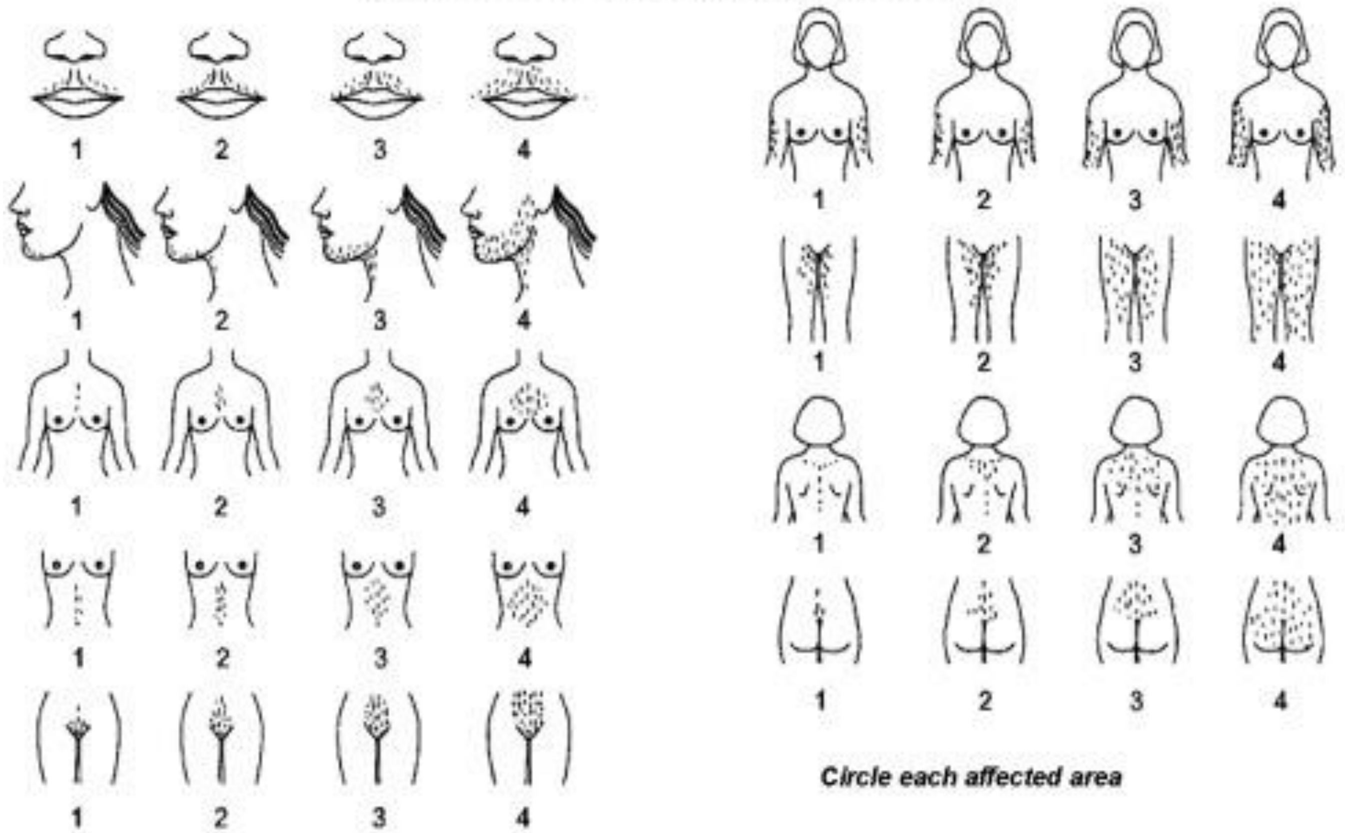

Circle each affected area

Total modified F-G score:

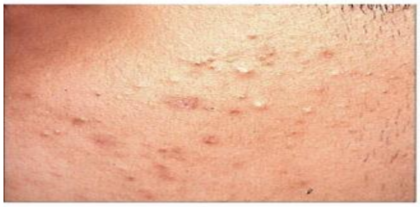

GRADE I MILD

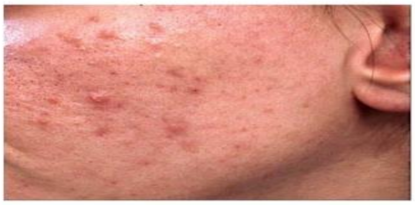

GRADE II MODERATE

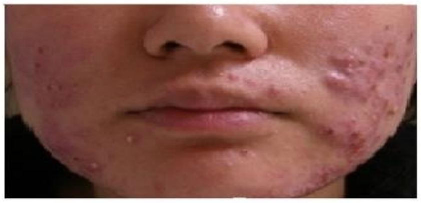

GRADE III SEVERE

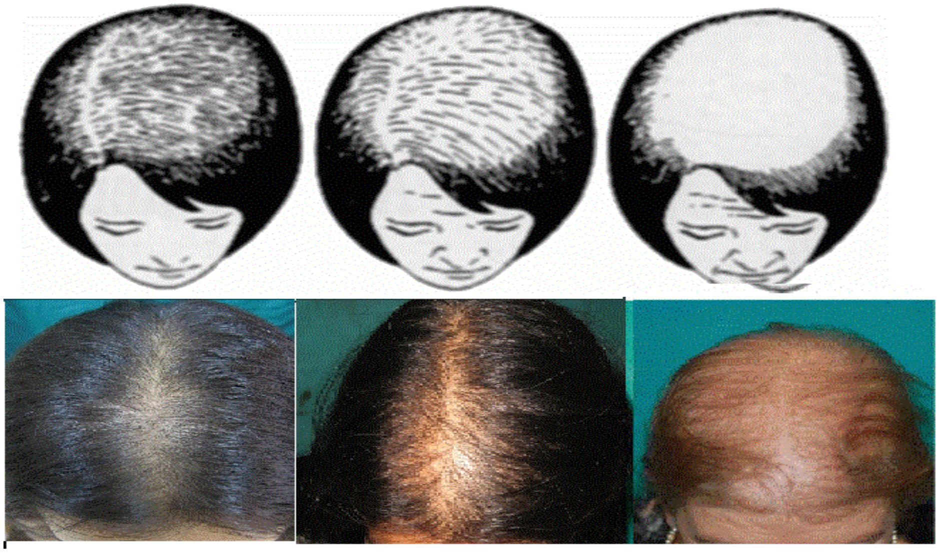

GRADE I GRADE II GRADE III

Section 3: Perception about self, health status and lifestyle

				Yes	No	
1.	What do you perceive your health as:			Yes	No	
		1.	Excellent			
		2.	Good			
		3.	Fair			
		4.	Poor			
2.	What do you feel about your present weight?	1	Would like to lose			
		2.	Would like to gain			
		3.	Satisfied			
3.	What do you perceive your lifestyle as?	1	Active			
		2.	Moderately active			
		3.	Sedentary			
4.	Do you suffer from any of the diseases?	1.	Tuberculosis			
		2.	Diabetes			
		3.	High Blood Pressure			
		4.	Thyroid			
		5.	Bone or joint related problem			
		6.	Other (please specify)			
5.	Do you get any leisure time?	1 ☐ Yes 2 ☐ No				
6.	If yes, how do you spend your leisure time?	1.	Rest			
		2.	Watch T.V.			
		3.	Sit /chat with family/friends			
		4.	Listen to music			
		5.	Talk on phone			
		6.	Work on computer/laptop			
		7.	Other (please specify)			
7.	Do you feel the need to work out?	1 ☐ Yes 2 ☐ No				
8.	If yes, do you plan your daily schedule for workout?	1 ☐ Yes 2 ☐ No				
9.	Do you undertake any physical activity?	1 ☐ Yes 2 ☐ No				
10.	If yes, which of the following activities do you undertake?					
		1.	Walking			
		2.	Jogging			
		3.	Yoga			
		4.	Aerobic exercises, Pilates,			
		5.	Sports (Please specify)			
		6.	Gym			
		7.	Cycling			
		8.	Swimming			
		17.	Other (please specify)			
11.	How many hours you spend in Univ /college?					

12.	What is the distance from your home?						
13.	On which floor is your class located?			Yes	No		If ground floor, skip to Q. 15
		1.	Ground floor				
		2.	First Floor				
		3.	Second Floor				
		17.	Other (please specify)				
14.	Do you use stairs or lift to your class/office?						
15.	On which floor is your house located?			Yes	No		If ground floor, skip to Q. 17
		1.	Ground floor				
		2.	First Floor				
		3.	Second Floor				
		17.	Other (please specify)				
16.	Do you climb stairs or lift to go to your home?						
17.	What is the distance of the most accessed market from home?						
18.	What is the usual mode of travelling used by you?	1	On foot				
		2.	Bus				
		3.	Metro				
		4.	Auto				
		5.	Car				
		6.	Other (please specify)				

19.	Do you carry weight and walk?	Weight	Never	Daily	Weekly	Occasionally	
		<1 kg					
		1-2 kg					
		3-5 kg					
		>5 kg					
20.	Do you have a television in your bedroom?	1 ☐ Yes 2 ☐ No					
21.	How many hours on an average (per day) do you spend sitting in front of a screen (laptop/television)?	Weekday-________hours<br><br>Weekend-________hours					

Section 4: Dietary intake pattern

1.	Food Habits	1 ☐ Vegetarian 2 ☐ Non-vegetarian<br>3 ☐ Ovo-vegetarian				
2.	How many meals do you consume in a day?			Yes	No	
		1.	Breakfast			
		2.	Mid-morning			
		3.	Lunch			
		4.	Tea time			
		5.	Dinner			
		6.	Bed time			
		17.	Other (please specify)			
3.	Do you eat or drink anything else in between two main meals?	1 ☐ Yes 2 ☐ No				

4.	If yes, how many in-between meals do you consume?	1 ☐ 1 meal 2 ☐ 2meals 3> ☐ [illegible]eals				
5.	What are food items generally consumed in between the main meals?			Yes	No	
		1.	Biscuit (Sweet/salty)			
		2.	Tea/Coffee			
		3.	Fried snacks (samosa, breadpakora, french fries)			
		4.	Chinese /Momos (steamed/ fried/ tandoori)			
		5.	Burger/ Sandwich			
		6.	Fruits (specify)			
		7.	Desserts (pasrty, sweets)			
		8.	Chocolates			
			Junk Food(Chips, pizza etc)			
6.	Do you skip main meals?	1 ☐ Yes 2 ☐ No				
7.	If yes, which main meal do you skip?	1.	Breakfast			
		2.	Mid-morning			
		3.	Lunch			
		4.	Tea time			
		5.	Dinner			
		6.	Bed time			
		7	Other (please specify)			
8.	What is the main reason for skipping main meals?	1.	Lack of appetite			
		2.	Lack of time			
		3.	Not appealing			
		4.	Trying to lose weight			
		5.	Other (specify)			
9.	Do you get your tiffin/lunch from home or eat out/ from the canteen?	1 ☐ Bring from home 2 ☐ Eat out/ Canteen				
10.	What do you eat if don't get lunch from home?	1.	Pizza/ Pasta			
		2.	Chole bhature			
		3.	Fried snacks (samosa, breadpakora, french fries)			
		4.	Chinese/ Momos (steamed/ fried/tandoori)			
		5.	Dosa/ idli/ vada			
		6.	Burger/ sandwich			
		7.	Desserts (pasrty, sweets)			
		8.	Chocolate			
11.	In usual routine, do you eat out?	1 ☐ Yes 2 ☐ No				

12.	If yes, what do you mostly eat?	1.	Pizza/ Pasta			
		2.	Chole bhature			
		3.	Fried snacks (samosa, breadpakora, french fries)			
		4.	Chinese/ Momos (steamed/ fried/tandoori)			
		5.	Dosa/ idli/ vada			
		6.	Burger/ sandwich			
		7.	Desserts (pasrty, sweets)			
			Other (please specify)			
13.	Are you influenced by the discounts offered by various online portals (facebook, groupon, mail) or the pamphlets circulated by various eating joints?	1 ☐ Yes 2 ☐ No				

Section 5: Clinical Examination:

1.	Height(cms)			
2.	Weight(kg)			
3.	Waist circumference(cms)			
4.	Hip Circumference(cms)			
5.	Systolic BP(mm of Hg)			
6.	Diastolic BP (mm of Hg)			
7.	Pulse / minute			
8.	Acne (Grade I,II,III)			
9.	Androgenic alopecia(Grade I,II,III)			
10.	Acanthosis Nigricans (Grade I,II,III)			
11.	Chest			
12.	CVS			
13.	Abdomen			
14	CNS			
15.	Secondary sexual characteristics			
16.	FG SCORE			
17.	Miscellaneous			

Section 6: Biochemical Assessment

1.	Hb	TLC	DLC					
		WBC		NEUT	Lymph	MONO	EO's	BASO
2.	Platelet	PBF	ESR			RBC		
3.	Urine Exam							
4.	CXR		15.ECG					

5.	Bil (mg/dl)	11.OT/AST	16.PT/ALT	20.ALP
6.	T. Pr (g/dl)	12.Alb	17.Urea (mg/dl)	21.Cr
7.	Na (mmol/L)	13.K	18.Ca (mg/dl)	22.P
8.	TG (mg/dl)	14.CHOL	19.HDL	23.LDL
9.	GTT (mg/dl)	O hr	1 hr	2 hr
10.	Uric Acid			

2. Hormones

T3 (ng/ml)	T4 (µg/dl)	TSH (mIU/ml)	LH (IU/ml)	FSH (IU/ml)	Prolactin (ng/ml)
T Testo (ng/ml)		DHEAS(µg/ml)	17-OHP (µg/ml)	Cortisol M	Cortisol E
	ODST (µg/ml)				
	Insulin (µg/ml)	0 hr	1 hr	2 hr	

3.Biomarkers

1.	TNF-Alpha	2.IL-10	3.IL-6
4.	IL-1β	5.hs CRP	6.Resistin
7.	Adiponectin		

Section 7: 24 hour dietary recall (2 weekdays+1 weekend)

DAY 1 (WEEKDAY)

Meal	Menu	Quantity (household measures)	Ingredients	Amount of ingredients (g)		Remarks		
				Household measures	Grams	Size of chapatti/Rice	Greasi ness	Consis tency

DAY 2 (WEEKDAY)

Meal	Menu	Quantity (household measures)	Ingredients	Amount of ingredients (g)		Remarks		
				Household measures	Grams	Size of chapatti/Rice	Greasi ness	Consis tency

DAY 1 (WEEKEND)

Meal	Menu	Quantity (household measures)	Ingredients	Amount of ingredients (g)		Remarks		
				Household measures	Grams	Size of chapatti/Rice	Greasi ness	Consist ency
